# Genetic diversity of Nile tilapia (*Oreochromis niloticus*) throughout West Africa

**DOI:** 10.1038/s41598-019-53295-y

**Published:** 2019-11-14

**Authors:** Curtis E. Lind, Seth K. Agyakwah, Felix Y. Attipoe, Christopher Nugent, Richard P. M. A. Crooijmans, Aboubacar Toguyeni

**Affiliations:** 1 WorldFish, Jalan Batu Maung, Batu Maung, 11960 Bayan Lepas Penang, Malaysia; 2 CSIRO Agriculture & Food, Castray Esplanade, Hobart, Australia; 3 Aquaculture Research and Development Center (ARDEC), CSIR-Water Research Institute, PO Box 139, Akosombo, Ghana; 4 Food and Agriculture Organization of the United Nations (FAO), Rome, Italy; 5 Wageningen University & Research, Animal Breeding and Genomics, Wageningen, The Netherlands; 6 Centre International de Recherche-Développement sur l’Elevage en zone Subhumide (CIRDES) 01 BP 454 Bobo-Dioulasso 01, Bobo-Dioulasso, Burkina Faso; 7 Université Nazi BONI (UNB) 01 BP 1091 Bobo-Dioulasso 01, Bobo-Dioulasso, Burkina Faso

**Keywords:** Genetic variation, Animal breeding, Freshwater ecology

## Abstract

Nile tilapia (*Oreochromis niloticus*) is a globally significant aquaculture species rapidly gaining status as a farmed commodity. In West Africa, wild Nile tilapia genetic resources are abundant yet knowledge of fine-scale population structure and patterns of natural genetic variation are limited. Coinciding with this is a burgeoning growth in tilapia aquaculture in Ghana and other countries within the region underpinned by locally available genetic resources. Using 192 single nucleotide polymorphism (SNP) markers this study conducted a genetic survey of Nile tilapia throughout West Africa, sampling 23 wild populations across eight countries (Benin, Burkina Faso, Côte d’Ivoire, Ghana, Togo, Mali, Gambia and Senegal), representing the major catchments of the Volta, Niger, Senegal and Gambia River basins. A pattern of isolation-by-distance and significant spatial genetic structure was identified throughout West Africa (Global *F*_ST_ = 0.144), which largely corresponds to major river basins and, to a lesser extent, sub-basins. Two populations from the Gambia River (Kudang and Walekounda), one from the western Niger River (Lake Sélingué) and one from the upper Red Volta River (Kongoussi) showed markedly lower levels of diversity and high genetic differentiation compared to all other populations, suggesting genetically isolated populations occurring across the region. Genetic structure within the Volta Basin did not always follow the pattern expected for sub-river basins. This study identifies clear genetic structuring and differentiation amongst West African Nile tilapia populations, which concur with broad patterns found in previous studies. In addition, we provide new evidence for fine-scale genetic structuring within the Volta Basin and previously unidentified genetic differences of populations in Gambia. The 192 SNP marker suite used in this study is a useful tool for differentiating tilapia populations and we recommend incorporating this marker suite into future population screening of *O. niloticus*. Our results form the basis of a solid platform for future research on wild tilapia genetic resources in West Africa, and the identification of potentially valuable germplasm for use in ongoing breeding programs for aquaculture.

## Introduction

The *Oreochromines* are a sub-family of the Cichlidae family of fishes native to Africa and parts of the Levant that have large commercial significance for aquaculture and capture fisheries^1^. The Nile tilapia, *Oreochromis niloticus*, has a broad natural distribution spanning from the Nile River basin southwards through the Eastern and Western Rift Valley lakes in East Africa, and westwards through the basins of Lake Chad, Niger, Benue, Volta, Gambia and Senegal rivers^1^. The species has been well studied from a taxonomic perspective. A complex sub-species structure and distribution of *O. niloticus* has been documented, especially across East Africa where a network of geographically and biologically diverse lakes have shaped its evolution. Seven sub-species of *O. niloticus* have been identified based on traditional taxonomic methods; all except one – O. *niloticus niloticus* - are distributed amongst the Rift Valley lakes of East Africa^1^.

Molecular studies have identified clear differentiation between west and east African populations of *O. niloticus*^2–5^, which can be generalized into three main groups: (i) Sudano-Sahelian populations, covering West Africa, (ii) Ethiopian Rift valley populations and (iii) Nile drainage and Kenyan Rift Valley populations^5,6^. A hierarchical pattern of genetic differentiation has been observed in *O. niloticus*, whereby the effects of major paleo-geographic and climatic events are the predominant factor at the macro-geographic scale, and present-day river systems are influential on genetic connectivity and patterns of genetic structure at smaller geographic scales^5^. The same authors have also suggested limited dispersal capabilities combined with non-random mating caused by social behaviours may also influence small scale population structure. Other studies indicate that isolated and previously undescribed *O. niloticus* populations in Kenya can show strong genetic differentiation from other nearby populations, and may constitute new wild genetic resources with potential importance for exploitation in commercial aquaculture activities^7^. Furthermore, studies of patterns of mitochondrial DNA variation have revealed additional complexities amongst *O. niloticus* populations, with evidence of historical hybridization occurring between closely related *O. aureus*^8^ and *O. leucosticus*^9^ species occurring in the western and eastern regions of Africa, respectively.

Although much attention has been given to the population characterization and genetic structure of *O. niloticus* in East Africa, relatively few studies have focused on populations throughout West Africa with high resolution. This is despite West Africa being the largest ichthyoregion naturally occupied by *O. niloticus*. Earlier studies have indicated genetic differentiation at broad scales (i.e. 1000’s of km)^3–5^. However, spatial coverage of these studies is limited and consequently knowledge of finer scale patterns of genetic structure of *O. niloticus* in West Africa remains uncertain.

*Oreochromis niloticus* is by far the most prominent amongst tilapia species produced by aquaculture, being the most farmed tropical fish species globally. Approximately 4.1 million tonnes of Nile tilapia were produced by aquaculture in 2017, having a value of US$7.6 billion^10^. West African *O. niloticus* populations have made a significant contribution towards the development of farmed genetic resources that underpin much of global production. For example, at least four of the eight founder populations (and likely to be five) of the highly successful Genetically Improved Farmed Tilapia (GIFT) strain of *O. niloticus* were either sourced directly from wild populations in Ghana and Senegal or cultured populations derived from stocks of Ghanaian origin^11^. The GIFT strain has undergone continuous selective breeding in Southeast Asia since the early 1990’s, and has been widely recognized as having substantial impact on tilapia aquaculture development across the region^12,13^. Additionally, a selective breeding program based on locally sourced wild *O. niloticus* is rapidly gaining popularity by aquaculture farmers in Ghana and bringing economic benefits and boosting primary productivity for the country^14–16^. Such successes demonstrate the potential value that can be derived from these wild genetic resources provided the appropriate management and technological approaches are applied.

Tilapia aquaculture is poised for rapid growth throughout Sub-Saharan Africa in the coming years. As tilapia aquaculture increases throughout Sub-Saharan Africa, an impetus to better understand the distribution of wild genetic resources and patterns of population structure throughout the natural distribution of *O. niloticus* is similarly increasing^6,17^. The historic popularity of Nile tilapia as a capture fishery and for aquaculture production has seen its repeated translocation and deliberate introduction to many places where it was not originally found. The genetic origin of such introductions are often vague or unknown, yet may have an important impact on the genetic composition of wild populations.

The advantages of investigating molecular genetic characteristics of wild Nile tilapia are two-fold. Firstly, understanding wild population structure can assist the management or safeguarding of wild genetic resources, particularly in the context of the potential impacts caused by aquaculture escapes. Secondly, the identification of genetically distinct and diverse wild populations is of potential benefit to aquaculture breeding programs, which require a broad genetic diversity in their founder populations to achieve sustained productivity improvements and avoid problems of inbreeding accumulation. By providing insights to both the management and use of tilapia genetic resources, molecular genetic data offers better understanding in a climate where large-scale aquaculture development in West Africa is growing and proper resource usage is critical.

This study presents the opportunity to understand patterns of genetic structure and factors affecting diversity across the region, to better document the natural Nile tilapia genetic resources in West Africa, and further build on previous research to understand the potential macro or micro geographic factors influencing their diversity. Using single nucleotide polymorphism (SNP) markers, we studied 23 populations across five river basins in West Africa to assess spatial patterns of genetic diversity and genetic differentiation of indigenous wild Nile tilapia populations. The specific aims of this study are to: (1) Characterize genetic diversity of Nile tilapia populations throughout West Africa using SNP markers; (2) Quantify the relative differences of genetic structure in Nile tilapia populations within versus among watershed basins throughout West Africa; (3) Explore the genetic characteristics of Nile tilapia populations not recognized as part of its natural distribution but are known locally to be sources of the species; and (4) Identify potential factors contributing to patterns of genetic diversity and differentiation in West African Nile tilapia populations. We anticipate this will provide a clearer insight towards what populations may be of potential value for conservation or further use in ongoing aquaculture expansion plans for the region.

## Material and Methods

### Origin and location of the sampling populations

**Figure 1:**
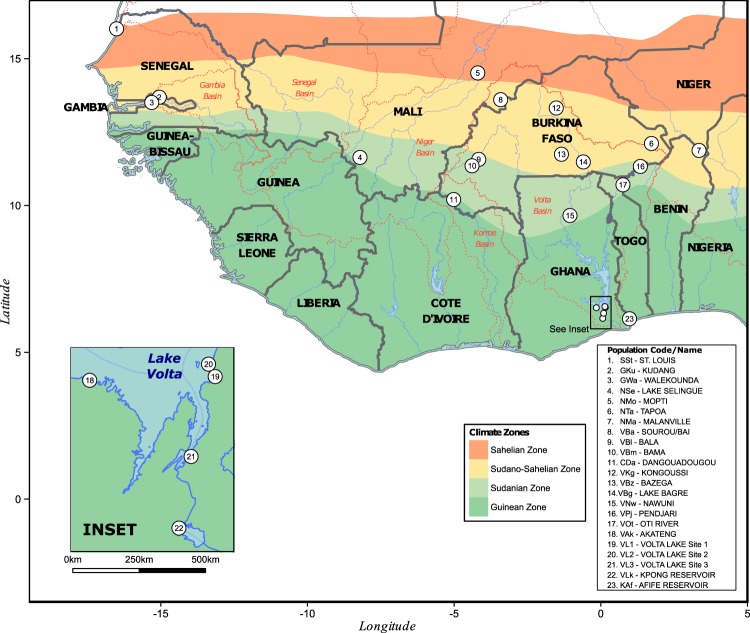
Sampling sites of Nile tilapia populations throughout West Africa. Dotted lines indicate boundaries of major river basins.

Twenty three wild *O. niloticus* populations were sampled from across eight countries (Benin, Burkina Faso, Côte d’Ivoire, Gambia, Ghana, Mali, Senegal and Togo) of West Africa, representing the major catchments of the Volta, Niger, Senegal and Gambia River basins (Fig. 1). Fine-scale patterns of genetic differentiation were targeted within the Volta Basin. Two to five population samples were taken from within several sub-basins, including Lake Volta, a large reservoir formed by the Akosombo Dam constructed in 1965, and each of its four main tributaries, namely, the Black Volta River (also known as the Mouhoun), the Red (Nazinon) and White Volta (Nakambé) Rivers and the Oti River (Table 1). One site from the Comoe Basin (Burkina Faso) and the Kloukpa River (Ghana) were also sampled, which are not recognized as being part of the natural distribution of *O. niloticus*^1^ but are known sources of the species locally.

**Table 1:** Location, major river basin and collection date of wild Nile tilapia populations sampled throughout West Africa.

Population	Code	Basin	Country	Latitude	Longitude	Collection Date
Saint Louis	SSt	SENEGAL	SENEGAL	16.0185	−16.509389	2011-12-23
Kudang	GKu	GAMBIA	GAMBIA	13.6877	−15.053380	2011-12-21
Walekounda	GWa	GAMBIA	GAMBIA	13.5092	−15.309772	2011-12-21
Lake Sélingué	NSe	NIGER	MALI	11.6381	−8.210422	2010-06-02
Mopti	NMo	NIGER	MALI	14.5158	−4.196269	2010-06-01
Tapoa	NTa	NIGER	BURKINA FASO	12.1115	1.726472	2010-02-15
Malanville	NMa	NIGER	BENIN	11.8757	3.348683	2010-01-17
Dangouadougou	CDa	COMOE	BURKINA FASO	10.2035	−5.01664	2010-03-25
Sourou/Bai	VBa	VOLTA - BLACK	MALI	13.6048	−3.413997	2010-05-31
Bala	VBl	VOLTA - BLACK	BURKINA FASO	11.5701	−4.161250	2009-12-30
Bama	VBm	VOLTA - BLACK	BURKINA FASO	11.3527	−4.390444	2009-12-30
Kongoussi	VKg	VOLTA - RED/WHITE	BURKINA FASO	13.3213	−1.519806	2010-01-03
Bazega	VBz	VOLTA - RED/WHITE	BURKINA FASO	11.7460	−1.342697	2010-01-31
Lake Bagre	VBg	VOLTA - RED/WHITE	BURKINA FASO	11.4844	−0.595472	2010-02-04
Nawuni	VNw	VOLTA - RED/WHITE	GHANA	9.6593	−1.050790	2010-03-25
Pendjari	VPj	VOLTA - OTI	BENIN	11.3310	1.348867	2010-01-19
Oti River - Mandouri	VOt	VOLTA - OTI	TOGO	10.7055	0.755317	2010-01-21
Akateng	VAk	VOLTA - LAKE	GHANA	6.5189	−0.152910	2010-09-23
Volta Lake - Site 1	VL1	VOLTA - LAKE	GHANA	6.5280	0.154450	2010-06-22
Volta Lake - Site 2	VL2	VOLTA - LAKE	GHANA	6.5584	0.138228	2010-03-25
Volta Lake - Site 3	VL3	VOLTA - LAKE	GHANA	6.3323	0.095460	2010-07-06
Kpong Reservoir	VLk	VOLTA - LAKE	GHANA	6.1572	0.065745	2010-07-09
Afife Reservoir	KAf	KLOUKPA RIVER	GHANA	6.1429	0.978297	2010-09-22

### Sample collection

A total of 1001 individual fish were sampled from 2009 to 2011. Fish were collected directly from commercial fishing boats or at the landing sites of known fishing areas. A caudal finclip approximately 1 cm × 2 cm was cut using forceps and a sharp, clean pair of scissors, and preserved in individually labeled vials using 95% ethanol. All the samples were stored at room temperature until later processing. Each sample site was geo-referenced using a handheld GPS receiver. Between 24 and 50 individuals were collected from each site, depending on availability. Sex of each fish was indeterminable from external examination at sampling, and it is assumed that an approximately even sex ratio of males to females were sampled. A summary of each sampling site, including geo-reference details, is described in Table 1.

### Ethics statement

Live experimental animals were not used for this study; thus the requirement for animal ethics approval was not applicable. Finclips were taken from dead fish caught by small-scale commercial fishers using standard gear and practices. Fishers were not commissioned by researchers of this study and study animals were purchased whole or sampled at the point of sale to the public on the day of catch.

### DNA extraction protocol

After collection, DNA extraction was performed using a commercially available kit (Promega WIZARD® Genomic DNA Purification Kit) as per the manufacturer’s instructions, and stored at −20 °C until later processing. DNA extraction was conducted at the laboratory of the International Centre for Research and Development on Livestock in Subhumid Zones (CIRDES), Burkina Faso. Samples were standardized to a DNA concentration of 50 ng ul^−1^ and shipped with dry ice by air to Wageningen University for genotyping.

### Single nucleotide polymorphism (SNP) genotyping

Multi-locus genotypes were generated from 192 SNP markers, selected from a suite of 384 SNP markers developed for *O. niloticus*^18^. Genotyping was performed in a 192 SNP multiplex assay using the Golden Gate Assay (Illumina), and deployed on a BeadXpress platform using Veracode technology. A total of 15 SNPs on the assay were located on chromosomes/linkage groups where there are known associations with sex determination (LG1, LG20, LG23) in Nile tilapia^19,20^, however, these SNPs used were not located in the vicinity of these sex-linked regions (data not shown) and therefore have minimal risk of biasing results of the study. A minimum call rate of 0.8 was used to score genotypes. A full list of the SNP markers used, the linkage group according to Guyon *et al*.^21^ and the dbSNP accession number of each SNP are given in the Supplementary Materials section.

### Statistical analyses

#### Genetic diversity

Number of polymorphic loci, observed heterozygosity and expected heterozygosity were calculated for each population using the R package *adegenet*^22^. Allelic richness of each population was calculated using the R package *hierfstat*^23^ based on a minimum sample size of 15 individuals, which accounts for differences in sample size and genotype failure. Tests for Hardy-Weinberg Equilibrium (HWE) were performed at each locus for each population using the *HWE.test.genind()* function of *adegenet*, based on the Chi-squared statistic. *P*-values of each test were adjusted within population according to the false discovery rate method of Benjamini and Hochberg^24^ using the *p.adjust()* function in R^25^, to an equivalent significance level of α = 0.05.

#### Effective population size (*Ne*)

Effective population size and its 95% confidence interval were estimated for each population using the linkage disequilibrium method of Waples^26^, as implemented in NeESTIMATOR v2.01^27^. A random mating model was applied, using a P_CRIT_ (lowest allele frequency) of 0.05.

#### Population differentiation and genetic distances

Wright’s *F*-statistics (*F*_ST_, *F*_IS_ and *F*_IT_) were estimated using ARLECORE, the console version of ARLEQUIN v3.5^28^, to determine the proportion of genetic variation partitioned within and among various levels of population sub-structure (i.e. individual, sub-population and total population). Pairwise *F*_ST_ between populations were also calculated using ARLECORE, with the significance of pairwise values tested using a non-parametric Monte Carlo approach (1000 permutations). A Bonferroni correction was applied to *P*-values using the *p.adjust()* function in R^25^ to control the likelihood of type I errors associated with multiple comparisons. To investigate the significance of major geographical factors such as river basins (watersheds) on genetic structuring in Nile tilapia, an hierarchical Analysis of Molecular Variation (AMOVA) was implemented using ARLEQUIN. Several models were tested based on different groupings of populations representing reasonable explanatory drivers of genetic differentiation. The aim here is to explore what groupings could better explain the observed genetic variation when partitioned amongst groups (*F*_CT_), amongst populations within groups (*F*_SC_) and amongst populations (*F*_ST_). The population were grouped by (1) major river basins; (2) major river basins and sub-basins (where present); and (3) climatic zones.

#### Isolation by distance test

To test if genetic differentiation patterns followed an isolation-by-distance model of population divergence, Mantel’s test for correlation between geographic distances (km) and genetic distances (*F*_ST_) was done based on 10 000 permutations using the *mantel.randtest()* function in *adegenet*. Geographic distances used are straight line distances between population sample sites calculated from longitude and latitude coordinates using the *earth.dist()* function of the R package *fossil*^29^.

#### Spatial principal component analysis (sPCA)

Spatial patterns of genetic variability in *O. niloticus* were further investigated through a spatial principal component analysis (sPCA) of population allelic frequencies^30^. This method allows the analysis of both variability in allele frequencies and spatial autocorrelation amongst populations. sPCA was conducted using the *spca()* function of *adegenet*^22^. Spatial information was incorporated into the analysis based on a Gabriel graph connection network of population sample sites. Monte-Carlo tests for the presence of significant global and local spatial structure were performed using the *global.rtest()* and *local.rtest()* functions of *adegenet*, respectively, based on 9999 permutations. Here, global structure refers to positive spatial autocorrelation (i.e. populations closer to each other are more similar) and local structure refers to negative spatial autocorrelation (closer populations are more dissimilar). Because of the potential sampling biases in allele frequencies caused by the presence of rare variants, the dataset was trimmed to use only those loci that had a minor allele frequency (MAF) greater than 0.01 across all individuals.

#### Genetic clustering - Bayesian approach

The Bayesian clustering approach implemented in the software STRUCTURE v2.3 was used to partition genotype data according a predefined number of groups (*K*) that conform to expectations of Hardy-Weinberg equilibrium and linkage equilibrium between loci^31,32^. An admixture model with correlated allele frequencies was chosen to estimate the group membership probability (*Q*) of each individual for *K* = 2 to *K* = 20. For each level of *K* ten replicates of 300 000 Markov-Chain Monte Carlo iterations were run after a 150 000 iteration burn-in period. Default parameters were used otherwise. To identify the most likely number of clusters the Evanno method^33^ was implemented using STRUCTURE HARVESTER^34^. CLUMPP^35^ was used to align clusters across replicate runs and calculate a mean *Q* estimate for each individual, which were visualized using DISTRUCT^36^.

#### Genetic clustering - Multivariate approach

An alternative approach to identifying genetically distinct clusters is through Discriminant Analysis of Principal Components (DAPC)^37^. DAPC creates synthetic variables (discriminant functions) that attempt to maximize differences between groups or genetic clusters whilst minimizing allele frequency variance within a cluster. It has a major advantage in that no population genetic assumptions (such as Hardy-Weinberg expectations) are required to describe patterns of genetic variation across individuals and clusters. Prior definitions of groups are required for DAPC, which were done using the *find.clusters()* function of *adegenet* to implement a *k*-means clustering algorithm. The function also generates a Bayesian Information Criterion (BIC) for each value of *k*, which was then used to identify the optimal value of *k*. Ideally the BIC will decrease until an optimal *k* is reached and then subsequently increase for greater values of *k*. DAPC was performed for values of *k* increasing from 2 to 20 using the *dapc()* function of *adegenet*, retaining 75 principal components and 6 discriminant functions for each value of *k*. The trimmed dataset containing loci with a MAF greater than 0.01 was also used for this analysis, for the same reasons described earlier.

## Results

### Dataset cleaning

A total of 192 SNPs were genotyped for all 1001 samples. Four samples were removed from the final dataset due to genotyping failure. Eight SNPs were monomorphic and seven SNPs had ≥15% missing genotypes, and were subsequently removed from the final dataset. A total of 177 SNP markers were used for the final analyses, unless otherwise stated. Fifty four SNP markers had a minor allele frequency (MAF) < 0.01.

### Summary statistics and genetic diversity

Genetic diversity was lowest in the Kudang, Walekounda (both located in the Gambia River basin), Lake Sélingué (western Niger basin) and Kongoussi (Red/White Volta basin) populations. Populations that showed the greatest level of genetic variation were from Malanville (eastern Niger basin) and two populations from the southern region of Ghana (Site VL2 from Lake Volta and the Afife Reservoir in southern Ghana). Total number of alleles within each population ranged from 257 to 345, which translated to an overall percentage of polymorphic loci by population ranging from 33.8 to 79.7 percent. Allelic richness (R_s_), a measure of the average number of alleles per locus taking into account potential biases due to differences in sample sizes, ranged from 1.30 to 1.62. Observed heterozygosity was very similar to expected heterozygosity in all populations when averaged across loci (Table 2), which indicates a general conformation to expectations of Hardy-Weinberg Equilibrium (HWE). This is supported by HWE tests by locus, whereby less than 5 percent of loci within each population showed a significant departure from Hardy-Weinberg expectations at all sites (*P* < 0.05), with the exception of Nawuni in northern Ghana (5.2 percent loci not in HWE) and the Afife Reservoir (15.1 percent).

**Table 2:** Genetic diversity summary statistics of wild Nile tilapia populations across West Africa (*H*_o_: observed heterozygosity; *H*_e_: expected heterozygosity; R_s_: allelic richness; HWE: Hardy-Weinberg equilibrium; *N*_e_: effective population size).

Population	Code	*n*	No. of alleles	Missing data %	*H*_o_	*H*_e_	Polymorphic loci %	R_s_	Loci not in HWE %	*N*_e_	*N*_e_ 95% CI
Saint Louis	SSt	49	331	2.93	0.19	0.18	72.4	1.53	2.1	172.0	110.5–356.7
Kudang	GKu	26	257	3.35	0.11	0.10	33.8	1.30	1.0	63.0	33.3–264.3
Walekounda	GWa	24	262	2.17	0.11	0.10	36.5	1.30	1.0	56.0	30.3–203.1
Lake Sélingué	NSe	44	268	1.48	0.12	0.12	39.6	1.33	2.1	75.4	50.9–131.5
Mopti	NMo	50	321	1.30	0.20	0.20	67.2	1.55	3.1	288.8	161.9–1082.9
Tapoa	NTa	29	331	3.57	0.19	0.19	72.4	1.54	2.1	59.9	42.2–97.7
Malanville	NMa	50	345	2.06	0.22	0.23	79.7	1.62	4.7	235.6	148.4–527.6
Dangouadougou	CDa	29	306	1.15	0.18	0.16	59.4	1.47	2.1	68.1	45.7–122.7
Sourou/Bai	VBa	50	317	1.19	0.20	0.20	65.1	1.55	2.6	143.2	102.3–228.6
Bala	VBl	50	322	3.92	0.22	0.20	67.7	1.55	3.1	161.5	107.3–304.9
Bama	VBm	30	306	1.30	0.19	0.19	59.4	1.51	2.1	174.5	91.1–1160.4
Kongoussi	VKg	45	258	1.47	0.12	0.11	34.4	1.30	2.1	351.6	115.8–∞
Bazega	VBz	45	305	1.48	0.20	0.19	58.8	1.51	2.1	57.5	46.5–73.8
Lake Bagre	VBg	50	296	1.81	0.19	0.17	54.2	1.47	3.1	91.5	68.4–132.8
Nawuni	VNw	50	315	1.72	0.19	0.19	64.1	1.53	5.2	183.6	120.8–356.8
Pendjari	VPj	49	316	3.44	0.18	0.17	64.6	1.50	3.6	139.7	93.8–255.6
Oti River - Mandouri	VOt	49	324	3.39	0.18	0.17	68.7	1.52	3.6	182.5	113.1–423.1
Akateng	VAk	50	322	1.97	0.21	0.20	67.7	1.55	3.6	102.5	78.6–143.3
Volta Lake - Site 1	VL1	30	306	1.42	0.20	0.19	59.4	1.53	3.1	80.7	55.9–136.9
Volta Lake - Site 2	VL2	49	344	2.91	0.20	0.20	79.2	1.58	4.7	156.9	106.6–280.4
Volta Lake - Site 3	VL3	49	314	1.32	0.18	0.18	63.5	1.51	2.6	63.3	51.4–80.5
Kpong Reservoir	VLk	50	338	2.65	0.20	0.19	76.0	1.56	3.1	110.3	82.2–162.0
Afife Reservoir	KAf	50	341	2.93	0.17	0.17	77.6	1.51	15.1	79.6	60.5–112.1

### Effective population size

Effective population sizes (*N*_e_) was in general positively correlated with genetic diversity measures. Over 50 percent of populations sampled had *N*_e_ in excess of 100 and none below 50. An exception to this pattern was the Kongoussi population in northern Burkina Faso, which showed the greatest *N*_e_ (351.6) yet had comparatively low heterozygosity and allelic richness (Table 2). Full details of the summary and genetic diversity statistics of each population are outlined in Table 2.

### Population differentiation

**Figure 2:**
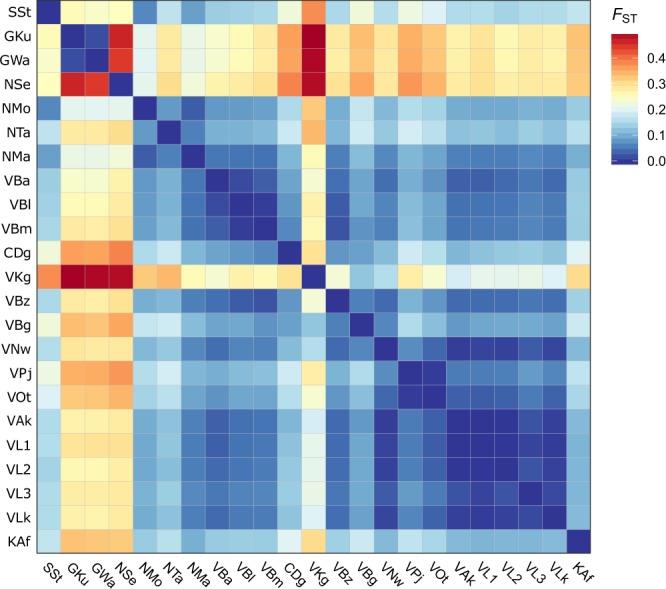
Heatmap of pairwise *F*_ST_ values amongst wild Nile tilapia populations.

Significant population differentiation was observed at the global level (*p* < 0.001), with 14.4% of overall genotypic variation attributable to differences among populations (i.e. *F*_ST._) when no hierarchical sub-groupings were considered other than the individual and population level (Table 3). When populations were grouped based on river basins using hierarchical AMOVA, regional patterns of genetic structure according to river basins or sub-basins begin to emerge. In some instances differences amongst regional grouping (*F*_CT_) accounts for up to 13.2 per cent of overall variation (Table 3). Grouping populations based on their climate zones could explain only 1.6% of overall variation. Population pairwise *F*_ST_ show significant differences amongst all populations with the exception of several groups of closely situated populations in the southern areas of Lake Volta (VAk, VL1 and VL2; and VL3 and VLk), two populations from the Gambia River (GKu and GWa), and two populations from the Oti River in the Volta Basin (VOt and VPj), which showed no significant differences to each other after Bonferroni correction. Clear patterns of genetic differentiation are observed between the Gambia River populations and all other populations sampled (Fig. 2). Populations from Lake Sélingué (western Niger River) and Koungoussi (upper Red/White Volta River) also showed high to moderate differences with all other sites, with pairwise *F*_ST_ ranging from 0.2 to 0.49 (Fig. 2).

**Table 3:** Analysis of Molecular Variation (AMOVA) of Nile tilapia based on various population groupings.

Grouping	Groups	Source of variation		
		Amongst populations	Amongst pop. within groups	Amongst groups
		*F*_ST_	*F*_SC_	*F*_CT_
none	*na*	0.144	—	—
Major Basins	*Volta*: [VBa, VBl, VBm, VKg, VBz, VB, VNw, VPj, VOt, VAk, VL1, VL2, VL3] *Niger*: [NSe, NMo, NTa, NMa] *Comoe*: [CDa] *Gambia*: [GKu, GWa] *Senegal*: [SSt] *Kloukpa*: [KAf]	0.190	0.094	0.107
Sub-basins	*Black* Volta: [VBa, VBl, VBm] *Red/White Volta*: [VKg, VBz, VBg, VNw] *Oti*: [VPj, VOt] *Lake Volta*: [VAk, VL1, VL2, VL3, VLk] *Niger*: [NSe, NMo, NTa, NMa] *Comoe*: [CDa] *Gambia*: [GKu, GWa] *Senegal*: [SSt] *Kloukpa*: [KAf]	0.161	0.070	0.097
Climatic zones	*Sahelian*: [SSt, NMo] *Sudano-Sahelian*: [GKu, GWa, NTa, NMa, VBa, VKg, VBg, VBz] *Sudanian*: [NSe, VBl, VBm, VPj, VOt] *Guinean*: [CDa, VAk, VL1, VL2, VL3, VLk, KAf]	0.157	0.143	0.016*

Populations are grouped based on major river basins, sub-basins, or climatic zones. Groups are indicated by square brackets. Population codes indicate all populations within a group. All statistics are highly significant (*p* < 0.001) unless otherwise indicated.

*P = 0.04.

### Spatial genetic patterns

**Figure 3:**
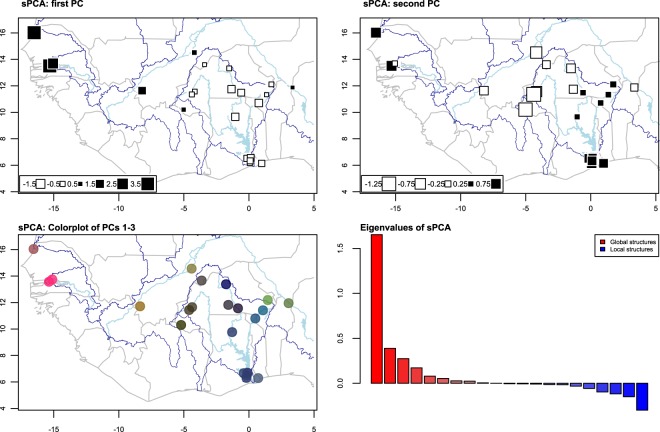
Spatial principal components analysis (sPCA) of Nile tilapia populations. The first two global principal components (PCs) are shown separately, where each square represents a population loading score. A combined illustration of all three PCs is shown through a colorplot, whereby the three loading scores are represented by colors according to the red, blue and green channels of the RGB color system.

Mantel’s test showed a significant, positive relationship between pairwise genetic distances (*F*_ST_) and geographic distances (see Supplementary Materials), indicating isolation-by-distance is a moderate influence on genetic differentiation at the regional scale (*r* = 0.521, *p* = 0.001). Consistent with this, sPCA revealed significant global spatial structure (*p* = 0.03) and non-significant local structure (*p* = 0.97) overall, indicating the genetic similarity of populations are positively correlated with their geographic proximity to each other. Within the Volta Basin, however, a significant yet much weaker relationship between genetic and geographic distances was present (*r* = 0.196, *p* = 0.029), indicating isolation-by-distance is a relatively minor factor shaping genetic differentiation amongst populations. Patterns of genetic differences and similarity across West Africa are evident when the lagged scores of the first three principal components of the sPCA are spatially plotted (Fig. 3). A “colorplot”, representing the lagged scores of the first three principal components of each population on the Red-Green-Blue colour space, highlights a genetic similarity within the Niger River basin populations, the Oti River and Lake Volta populations, the Red/White Volta and the Black Volta River populations, and the Gambia River populations (Fig. 3).

### Individual genetic clustering

**Figure 4:**
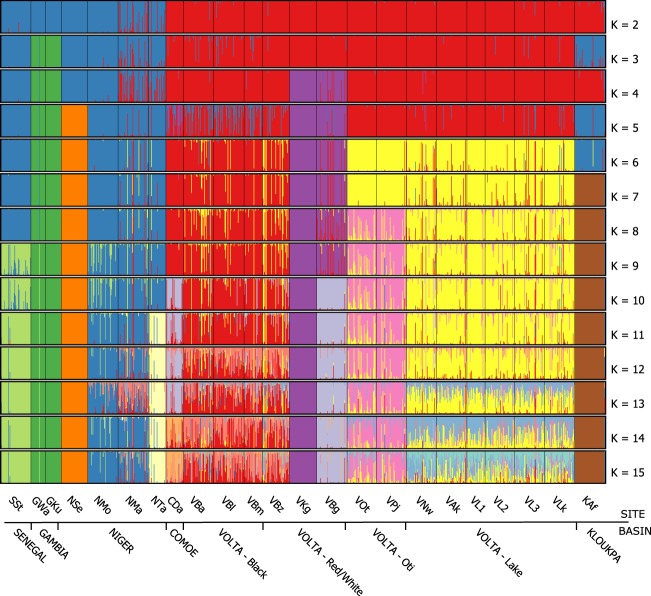
Bar plots representing individual-based clustering analyses of Nile tilapia throughout West Africa performed for differing number of clusters (*k*) using discriminant analysis of principal components (DAPC). Each vertical line represents an individual, and each color represents the membership probability of an individual to a cluster for a given value of *k*. Black bars separate different populations. Upper labels refer to population code and lower labels indicate river basin (or sub-basin).

**Figure 5:**
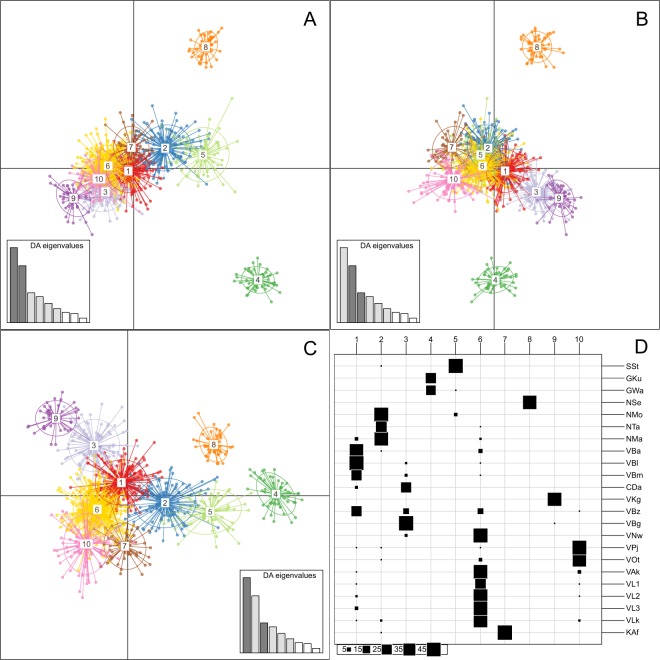
DAPC scatterplot of Nile tilapia SNP genotypes displaying (**A**) principal components 1 and 2; (**B**) principal components 2 and 3; and (**C**) principal components 1 and 3, for *k* = 10 clusters. Clusters are represented by colours and inertia ellipses, and individuals are shown as dots. Colours used for each cluster correspond to the same colours as those used for clusters in Fig. 4. (**D**) Number of individuals of each population assigned to different cluster groups. Population codes are listed in Table 1.

Bayesian (STRUCTURE) and multivariate (DAPC) based approaches revealed highly consistent patterns of individual genetic clustering for differing values of *k* ranging from 2 to 15. For clarity, only DAPC results are shown. Plots of individual posterior probabilities for assignment to a given cluster show that patterns of genetic differentiation based on river basin are apparent at various values of *k* (Fig. 4). The most likely number of clusters (*k*) for the Bayesian approach was four, based on the Delta K method^33^, although a secondary peak in Delta K is also seen for *k* = 13 which could indicate a more fine scale genetic structuring. Changes in BIC showed that an optimum value of *k* to use for DAPC is likely to fall between 10 and 15. Detailed changes in BIC and Delta K, and the comparison between DAPC and STRUCTURE results can be found in the Supplementary Materials. Clustering at the lowest level (*k* = 2) shows a distinction between the Volta Basin populations and all others, particularly for the DAPC analysis (Fig. 4). As the number of clusters (*k*) increases, individual clustering follows closely according to river basin. Within the Volta Basin, four genetic clusters can be observed and can be described as a Lake Volta cluster (including Nawuni, which lies on the White Volta River), an Oti River cluster, a Black Volta cluster and a Red/White Volta cluster. The relative differences amongst clusters identified with DAPC is visualized for *k* = 10 in Fig. 5. It highlights the largest genetic differences among all clusters are from two clusters comprised of individuals that are from Lake Sélingué (Niger River) and from the two populations of the Gambia River.

## Discussion

The multiple analyses conducted in this study all corroborate a pattern of spatial genetic structure and differentiation of Nile tilapia populations across West Africa. The broad scale isolation-by-distance patterns are consistent with the significant spatial correlations of population allele frequencies identified by sPCA. Genetic differentiation of *O. niloticus* across West Africa can generally be explained by river basins, and to some extent, sub-basins, with gene flow amongst populations following patterns of isolation-by-distance. However, genetic similarity in populations that are over 1400 km apart connected by the Niger River (e.g. Malanville and Mopti) provides evidence that sufficiently high gene flow along a large, continuous waterway can occur over genetically relevant timescales. In contrast, within a much shorter distance around the Red and White Volta River regions we see genetic heterogeneity among several populations (Bazega, Lake Bagre and Nawuni), indicating population differences can persist across relatively nearby geographies.

Our study reveals additional population complexity that has not been documented previously. The most divergent populations amongst those sampled were from Lake Sélingué in the Niger Basin and from the Gambia River, which showed strong differentiation to all other populations. Unlike Rognon & Guyomard^8^, who observed *O. niloticus* populations from Senegal to be most divergent from other West African populations, we observe a genetic similarity between populations from Senegal and those from Upper and mid-Niger River. This is consistent with the belief that following the last pluvial, around 12,000 to 7,500 years ago, the Senegal and Gambia Rivers were recolonized by freshwater fauna from the Niger Basin^38^. Similarly, the Black Volta and the Pendjari Rivers are likely to have been tributaries of the Niger River in the past^38^, which may explain the moderate genetic differentiation observed among sampling sites covering the three main tributaries of the Volta Basin.

We find genetic diversity using SNPs to be similar across the majority of populations sampled, with the exception of several populations exhibiting lower genetic diversity than others. Most populations showed greater SNP heterozygosity than previously reported for the Volta Basin^18^ and in two selectively bred tilapia populations^39^, albeit using SNP arrays not necessarily optimized for informativeness in West Africa. Lower genetic diversity in the Gambia River and the far western region of the Niger River is consistent with patterns often seen at a species range limit or isolated populations. Rognon & Gouyomard^8^ found decreased enzyme diversity in Nile tilapia samples from the Senegal River and cited founder effects at the margins of the distribution as the likely explanation. Founder effects may explain the pattern of genetic differentiation and reduced diversity identified in the Gambia River. In contrast, however, we found relatively high levels of SNP diversity in the Senegal River population, which may indicate this region is supporting larger, more stable populations compared to the nearby Gambia River. This is corroborated by differences in *N*_e_, which in the absence of gene flow would lead to differing rates of diversity loss through genetic drift. Alternatively, the Senegal River may have had more frequent, intermittent historical connectivity to the Niger Basin^40^, possibly modulating the impacts of genetic drift on diversity loss. Two populations at Lake Sélingué in the Upper Niger River, and Kongoussi in the Upper Red Volta River also show a substantially lower percentage of polymorphic loci than others. This may be a result of their geographic location in the upper reaches of their respective river systems causing relative isolation. Consequently, limited gene flow between other populations or increasing exposure to seasonal fluctuations and intermittence in water flow could explain a likely increase the rate of genetic diversity loss caused by genetic drift.

Rapid population expansions as a result of the new water bodies created by dams can potentially have pronounced effect on the genetic properties of a population^41^. Lake Volta, created after the completion of the Akosombo Dam in 1965, is the largest man-made lake in the world by surface area and spans 8,502 km^2^. Despite such vast areas and the likely enormous expansion of Nile tilapia populations throughout the lake after its formation we detect no evidence for departures from Hardy-Weinberg Equilibrium (HWE) and little genetic differentiation amongst any Lake Volta populations, with some over 400 km apart. This could mean that there has either been a limited founder effect on Nile tilapia populations as a result of the formation of Lake Volta and that genetic structuring of these populations has not changed. Alternatively, any genetic differentiation prior to or post Akosombo Dam construction has been homogenized by sufficient gene flow across the lake and equilibrium of allele frequencies has been reached. Across all sites, only the population from the Afife Reservoir exhibited notable HWE departures. This site is located adjacent to the mouth of the Volta River, in the Kloukpa River basin, and is not thought to part of the natural distribution of Nile tilapia^1^. A departure from HWE coupled with moderate genetic differentiation and relatively low effective population size seen in the Afife Reservoir is a possible indication of founder effects due to being recently established, likely from nearby Volta River populations.

Previous population genetic studies of Nile tilapia have identified major breaks across its natural distribution, delineating three macro-geographic groups: (i) Sudano-Sahelian populations, covering West Africa, (ii) Ethiopian Rift valley populations and (iii) Nile drainage and Kenyan Rift Valley populations^4,5^. Our study identifies a greater overall *F*_ST_ amongst populations within the Sudano-Sahelian region than previous estimates based on microsatellite markers (R_ST_ = 0.09)^5^. This could be due to a greater sampling coverage, particularly the inclusion of populations from the Gambia River, which are highly differentiated from all other populations sampled. This, however, may also be due to differences in expected heterozygosity derived from highly polymorphic microsatellite markers compared to dimorphic SNP markers, which can influence the upper bounds of possible *F*_ST_ values that can be obtained^42^. Our study presents the first estimate of population diversity based on SNP markers in West Africa, providing an important baseline as SNP increasingly become the marker of choice for population studies and for commercial aquaculture breeding programs.

Climatic fluctuations in the Sahel region can be dramatic throughout the year, especially compared to the tropical regions of southern Ghana, which may exert greater selective pressure for commercially significant traits such temperature and salinity tolerance. Temperature ranges from 12 to 47 °C throughout the year in Burkina Faso^43^, contrasting to temperatures that rarely extend beyond 21 to 34 °C in southern Ghana^44^, which highlights the vastly different thermal amplitudes within the natural range of *O. niloticus*. Although hierarchical AMOVA based on grouping of populations according to their climatic zone did not explain as much variation as groupings based on river basins, the presence of pockets of genetically isolated populations throughout the Sahel region identified in this study, such as Lake Sélingué and Kongoussi, are of particular interest in this context. Notably, the population at Kongoussi may likely experience fluctuations in environmental conditions substantially greater than the populations in the main rivers due to low water exchange, extended periods of zero rainfall and its shallowness across that region. Genetically unique populations that are potentially valuable genetic resources have been recently discovered in other regions of Nile tilapia’s natural distribution, notably in East Africa^7^. Results such as this provide a basis for more in-depth investigation into whether genetically distinct populations represent potential genetic resources for future utilization in commercial breeding programs. Future genetic studies would be well placed to target greater sampling intensities and finer-scale geographic coverage around areas where genetically distinct populations are present. Complementary to this, research focusing on understanding whether such differentiation confers to any physiological differences at the population level would be especially valuable.

In addressing questions of genetic diversity and genetic structuring of West African Nile tilapia populations, the potential implications on the growing tilapia aquaculture industry throughout the region must be considered. We show there are high levels of genetic diversity amongst populations located across most major water basins, and identify some populations that are highly differentiated from others, indicating the presence of genetically unique populations. Our results suggest there is ample genetic diversity throughout wild Nile tilapia populations in West Africa to support the establishment and long-term development of selective breeding programs for aquaculture in the region. The consequence, however, of a large aquaculture industry based on selectively bred, domesticated populations, is that widespread use and distribution of these animals is inevitable. This may have potential impacts on natural populations when farmed animals unintentionally escape, and is a growing issue in other parts of Africa^17,45–48^. Knowledge of local population genetics developed through studies such as this (possibly utilising more targeted SNP genotyping tools) have an important role to detect, analyse and help manage the potential the impacts of likely escapes. Recent work identifying species-specific SNP markers among closely related tilapia species would also be of value where geographical overlaps occur, and there is potential for hybridization^49^.

## Conclusions

This study identifies clear genetic structuring and differentiation amongst West African Nile tilapia populations, which concur with broad patterns found in previous studies^4,5,8^. In addition, this study provides new evidence for fine-scale genetic structuring within the Volta Basin and for further genetically differentiated populations in Gambia. Spatial genetic patterns and individual clustering methods indicate genetic structure can be largely explained by major rivers and sub-basins throughout the region. The 192 SNP marker suite used in this study is an effective tool for differentiating tilapia populations and we recommend incorporating this marker suite into future population screening of *O.niloticus*, potentially in conjunction with other species-specific SNPs. Our results form the basis of a solid platform for future research of wild tilapia genetic resources in West Africa, and the identification of potentially valuable germplasm for use in aquaculture breeding programs.

## Supplementary information

Dataset 1

Supplementary Info 2

Supplementary Info 3

## Data Availability

The full dataset and metadata from this publication is available from the Dryad Digital Repository (10.5061/dryad.bk3j9kd6m).
