# Supplementary material for: Genetic diversity of Nile tilapia (Oreochromis niloticus) throughout West Africa
Source: Sci Rep. 2019 Nov 14;9:16767. doi: 10.1038/s41598-019-53295-y (PMC6856548; doi:10.1038/s41598-019-53295-y)

Supplementary Information 2: Mantel's test between geographic and genetic distance (F_{ST}) among *O. niloticus* populations in West Africa.

From: Lind, C.E., Agyakwah, S.K., Attipoe, F.Y., Nugent, C., Crooijmans, R.P.M.A., Toguyeni, A. Genetic diversity of Nile tilapia (*Oreochromis niloticus*) throughout West Africa. Scientific Reports

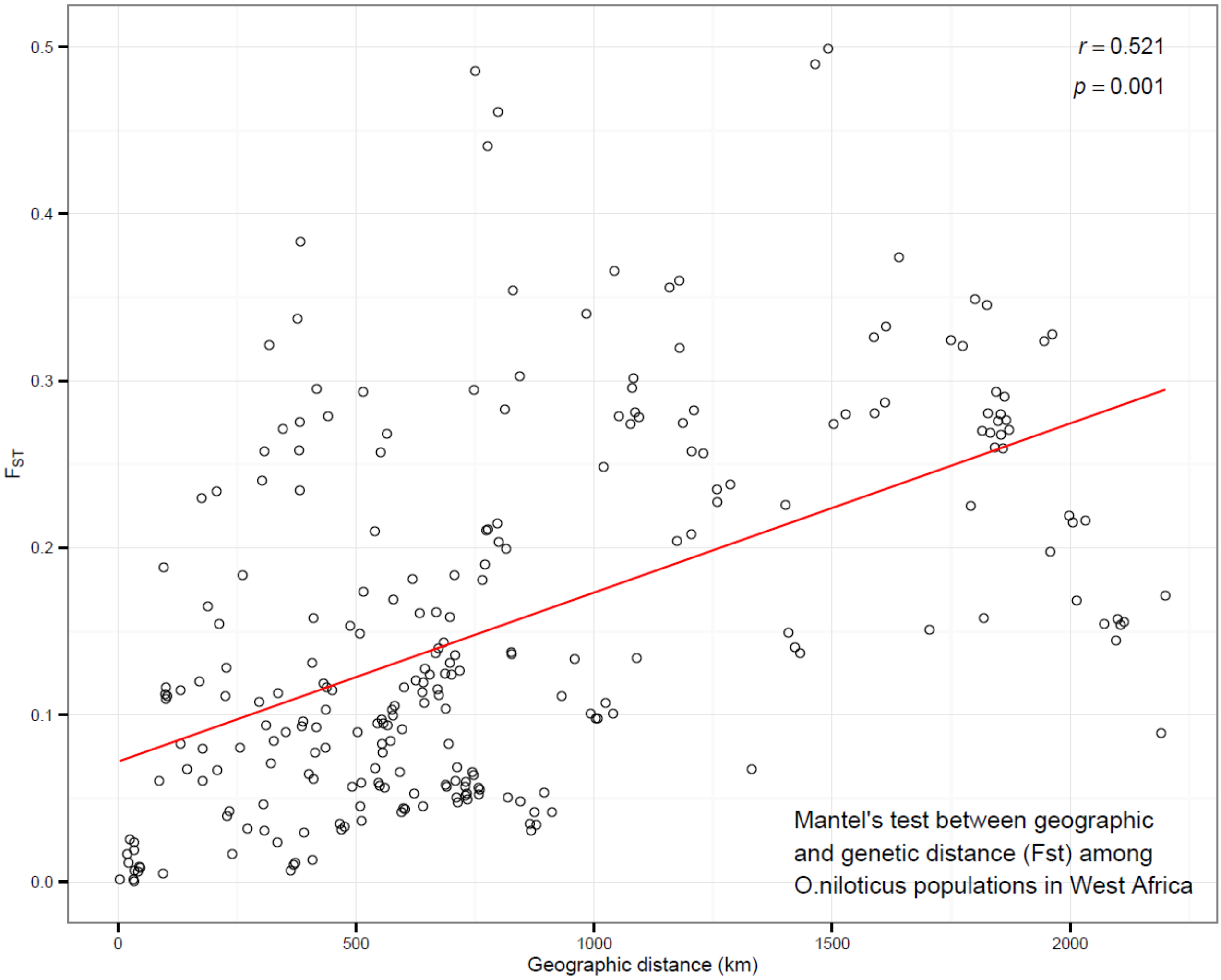

Supplement: Supplementary file 2 — Supplementary Info 2 [file 41598_2019_53295_MOESM2_ESM.pdf]
