# Supplementary material for: Genetic diversity of Nile tilapia (Oreochromis niloticus) throughout West Africa
Source: Sci Rep. 2019 Nov 14;9:16767. doi: 10.1038/s41598-019-53295-y (PMC6856548; doi:10.1038/s41598-019-53295-y)

Supplementary Information 3: Delta k and BIC vs number of clusters used for STRUCTURE and DAPC analysis of wild *O. niloticus*, respectively.

From: Lind, C.E., Agyakwah, S.K., Attipoe, F.Y., Nugent, C., Crooijmans, R.P.M.A., Toguyeni, A. Genetic diversity of Nile tilapia (*Oreochromis niloticus*) throughout West Africa. Scientific Reports

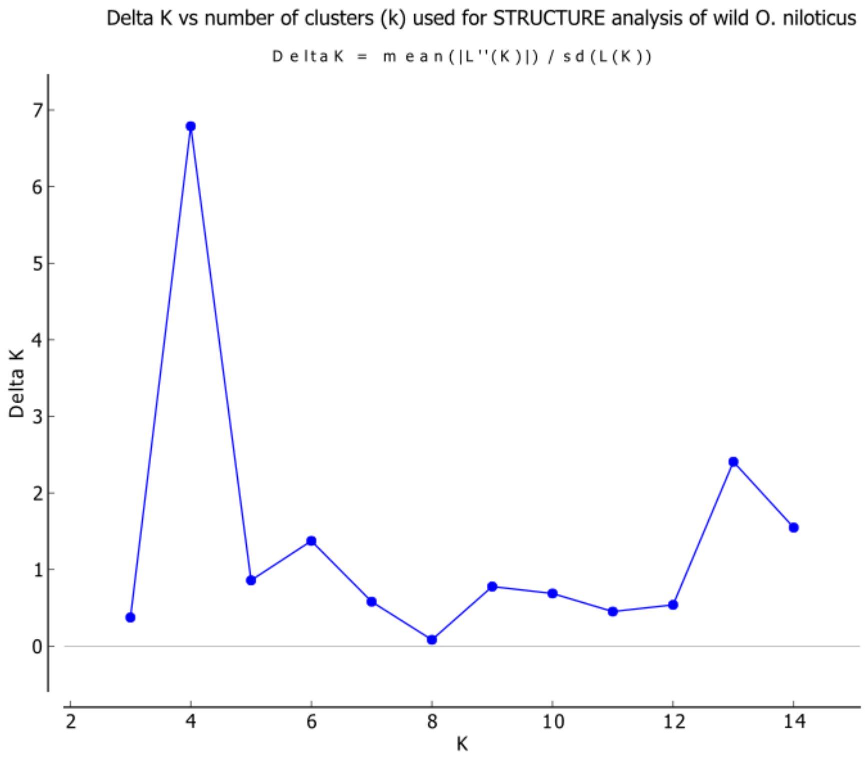

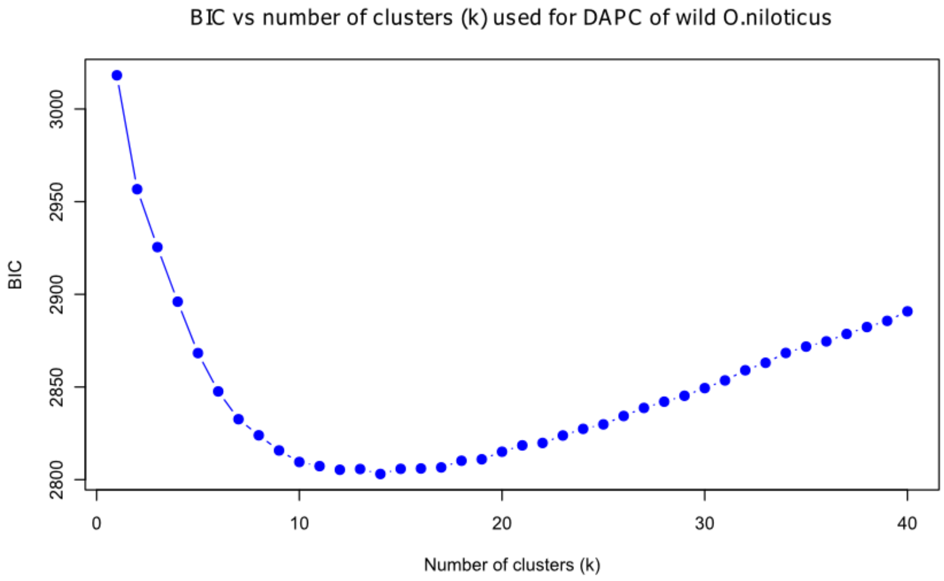

Supplement: Supplementary file 3 — Supplementary Info 3 [file 41598_2019_53295_MOESM3_ESM.pdf]
